# Interplay between the Reorientational Dynamics of the B_3_H_8_^–^ Anion and the Structure in KB_3_H_8_

**DOI:** 10.1021/acs.jpcc.0c10186

**Published:** 2021-02-16

**Authors:** M. S. Andersson, J. B. Grinderslev, X.-M. Chen, X. Chen, U. Häussermann, W. Zhou, T. R. Jensen, M. Karlsson, T. J. Udovic

**Affiliations:** † Department of Chemistry and Chemical Engineering, Chalmers University of Technology, Göteborg SE-412 96, Sweden; ‡ NIST Center for Neutron Research, National Institute of Standards and Technology, Gaithersburg, Maryland 20899-6102, United States; § Interdisciplinary Nanoscience Center (iNANO) and Department of Chemistry, Aarhus University, DK-8000 Aarhus, Denmark; ∥ School of Chemistry and Chemical Engineering, Henan Key Laboratory of Boron Chemistry and Advanced Energy Materials, Henan Normal University, Xinxiang, Henan 453007, China; ⊥ College of Chemistry and Green Catalysis Center, Zhengzhou University, Zhengzhou, Henan 450001, China; # Department of Materials and Environmental Chemistry, Stockholm University, SE-10691 Stockholm, Sweden; % Department of Materials Science and Engineering, University of Maryland, College Park, Maryland 20742-2115, United States

## Abstract

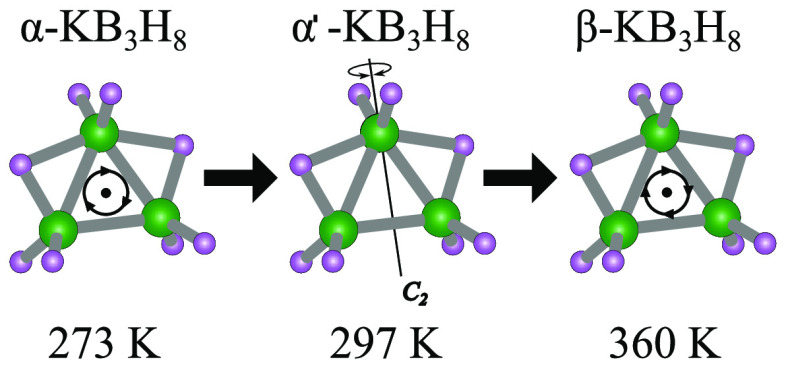

The structure and reorientational dynamics of KB_3_H_8_ were studied by using quasielastic and inelastic neutron scattering, Raman spectroscopy, first-principles calculations, differential scanning calorimetry, and *in situ* synchrotron radiation powder X-ray diffraction. The results reveal the existence of a previously unknown polymorph in between the α′- and β-polymorphs. Furthermore, it was found that the [B_3_H_8_]^−^ anion undergoes different reorientational motions in the three polymorphs α, α′, and β. In α-KB_3_H_8_, the [B_3_H_8_]^−^ anion performs 3-fold rotations in the plane created by the three boron atoms, which changes to a 2-fold rotation around the *C*_2_ symmetry axis of the [B_3_H_8_]^−^ anion upon transitioning to α′-KB_3_H_8_. After transitioning to β-KB_3_H_8_, the [B_3_H_8_]^−^ anion performs 4-fold rotations in the plane created by the three boron atoms, which indicates that the local structure of β-KB_3_H_8_ deviates from the global cubic NaCl-type structure. The results also indicate that the high reorientational mobility of the [B_3_H_8_]^−^ anion facilitates the K^+^ cation conductivity, since the 2-orders-of-magnitude increase in the anion reorientational mobility observed between 297 and 311 K coincides with a large increase in K^+^ conductivity.

## I Introduction

Over the past decades, metal borohydrides have undergone a remarkable transformation from a small family of compounds, primarily investigated for their hydrogen-storage properties, to a family of materials with a wide range of interesting properties, such as superionic conductivity, luminescence, and magnetism.^1−3^ The wide array of properties found in metal borohydrides is linked to the flexible structural and compositional nature of these compounds. One of the important borohydride groups are the octahydridotriborates, i.e., [B_3_H_8_]^−^, which are used not only as precursors for synthesis of higher borates, such as B_4_H_10_,^4^ but also as ionic liquids and in neutron capture therapy drugs.^5,6^ Recent chemical bonding analysis revealed that the [B_3_H_8_]^−^ anion is a σ-aromatic species.^7^ In addition to these properties, octahydridotriborates have received attention due to their high hydrogen density and generally low decomposition temperatures. While the decomposition of many of the octahydridotriborates involves the release of unwanted volatile boranes such as B_2_H_6_ and B_5_H_9_,^8,9^ KB_3_H_8_ releases mostly pure hydrogen after adding a metal chloride (e.g., ZrCl_4_) to the dehydrogenation process, thus making it a candidate for hydrogen storage.^8^

**Figure 1:**
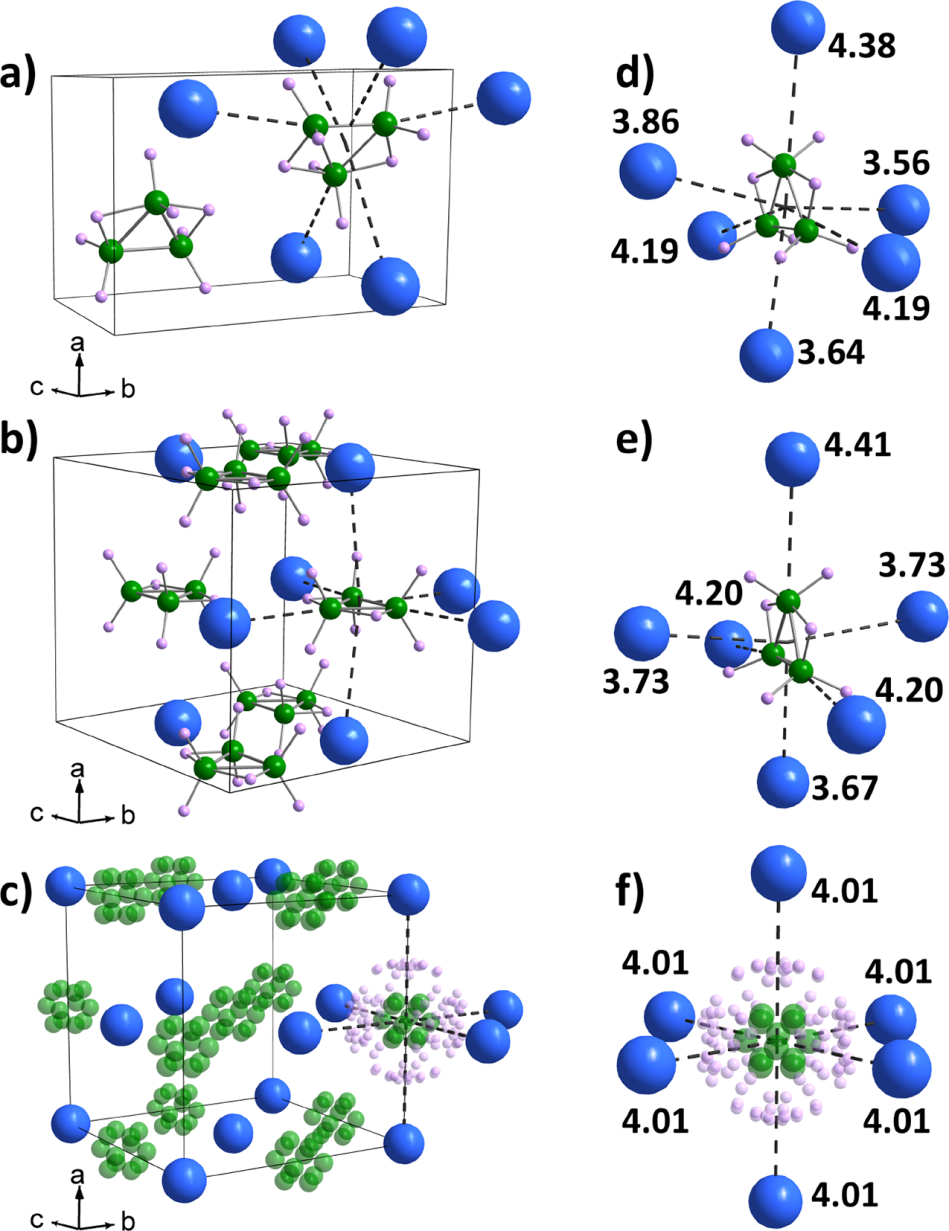
Crystal structures as determined in ref (10): (a) α-KB_3_H_8_, (b) α′-KB_3_H_8_, and (c) β-KB_3_H_8_. (d–f) Local environment of the [B_3_H_8_]^−^ anion for α-, α′-, and β-KB_3_H_8_, respectively. The numbers in (d–f) indicate the distances in Å of the K cations to the center of the [B_3_H_8_]^−^ anion. Color scheme: K (blue), B (green), and H (purple).

In a recent X-ray diffraction study of the temperature-induced structural evolution of KB_3_H_8_, it was found that KB_3_H_8_ exhibits three polymorphs (α-KB_3_H_8_, α′-KB_3_H_8_, and β-KB_3_H_8_).^10^ In particular, in the temperature interval 233–288 K, Bragg reflections of the monoclinic α-polymorph start merging, and at 288 K, it undergoes a second-order polymorphic transition to the orthorhombic α′-polymorph. Upon further heating, the α′-polymorph exhibits a first-order polymorphic transition to the cubic β-polymorph at 303 K. The study also showed that the [B_3_H_8_]^−^ anion is in an ordered state in the lower-temperature α- and α′-polymorphs, while transforming to an orientationally disordered state in the high-temperature β-polymorph (see Figure 1).^10^ The anion disorder suggests that it might be undergoing rapid reorientations similar to what has been observed in other metal borohydrides such as MBH_4_ (M = Li, Na, K, and NH_4_) and the closo-polyborates M_2_B_10_H_10_ and M_2_B_12_H_12_ (M = Li and Na).^11−13^ While the reorientations are primarily a local property, it can drastically affect some global properties such as ionic conductivity and the crystal structure of the material.^12−14^ Therefore, understanding the reorientational dynamics is of vital importance for fully understanding the properties of these technologically important materials.

In this study, we used quasielastic neutron scattering (QENS) to determine the reorientational dynamics of the different polymorphs of KB_3_H_8_. The results show that the [B_3_H_8_]^−^ anion performs 3-fold rotations in the plane created by the three boron atoms of the anion in the α-polymorph, which changes to a 2-fold rotation around the *C*_2_-axis in the α′-polymorph. In the β-polymorph, the anion undergoes 4-fold rotations, which is incompatible with the observed global cubic structure of β-KB_3_H_8_. This suggests that the structure is distorted and of lower symmetry on a local level, allowing each individual anion to adopt one of a variety of spatial orientations and preferred planes of rotation within the structure. Furthermore, we used inelastic neutron scattering (INS) and Raman spectroscopy to study the vibrational properties of the compound.

## II Experiments

### Synthesis, X-ray Diffraction, Differential Scanning Calorimetry, and Raman Spectroscopy

A polycrystalline powder sample of KB_3_H_8_ was synthesized and prepared as described elsewhere.^10,15−17^ It consisted of crystalline fractions KB_3_H_8_ (99 wt %) and KBH_4_ (1 wt %), as determined from powder X-ray diffraction, while ^11^B nuclear magnetic resonance revealed the presence of minor amounts of K_2_B_12_H_12_ and “BO_3_” and “BO_4_” species.^10^*In situ* time-resolved synchrotron radiation powder X-ray diffraction (SR PXD) data were collected at the beamline I11 at the Diamond Light Source, Oxford, UK, on a wide-angle position-sensitive detector based on Mythen-2 Si strip modules with λ = 0.824958 Å and at the MS-powder beamline at the Swiss Light Source (SLS), PSI, Switzerland, on a Mythen detector, λ = 0.70870 Å.^18^ The samples were packed in 0.5 mm borosilicate capillaries, sealed in an argon atmosphere, and heated by using an Oxford cryostream. Indexing and structural solutions were done by using the software FOX, and subsequent Rietveld refinements were performed in Fullprof.^19,20^ The [B_3_H_8_]^−^ anion was treated as a rigid body during structural optimization and Rietveld refinement. Pseudo-Voigt profile functions were used to fit the diffraction peaks, and the backgrounds were described by linear interpolation between selected points.

Differential scanning calorimetry (DSC) measurements with thermogravimetric analysis (TGA) were performed with a Netzsch (STA 449 F1 Jupiter) TGA-DSC^21^ under He flow by using about 5 mg of material in a sealed Al sample pan. Raman spectra between 303 and 327 K were collected on a small amount of sample contained inside a sealed quartz capillary by using a Renishaw inVia Raman microscope with a laser wavelength of 532 nm.

### Neutron Scattering Experiments

The neutron experiments were performed at the NIST Center for Neutron Research by using the high flux backscattering spectrometer (HFBS),^22^ the time-of-flight disc chopper spectrometer (DCS),^23^ and the filter analyzer neutron spectrometer (FANS).^24^ HFBS and DCS were used to study the reorientational dynamics of the [B_3_H_8_]^−^ anion in KB_3_H_8_, while FANS was used to study the vibrational properties of KB_3_H_8_. The neutron data were reduced and analyzed by using DAVE.^25^ The three spectrometers cover different energy ranges. HFBS covers an energy range of about 1–30 μeV, while DCS covers an energy range of 10 μeV–10 meV depending on the incoming neutron wavelength. FANS covers an energy range of about 5–250 meV.

For HFBS, ∼50 mg of polycrystalline powder sample was evenly distributed in an aluminum foil sachet, and the thickness was kept thin to minimize the significant absorption of neutrons from the ^10^B present in natural boron so as to allow measurements using a transmission geometry. The sachet was rolled into an annular shape and placed into a sealed cylindrical aluminum can. For DCS and FANS, a larger amount (≈0.7 g) of material was evenly distributed into another Al sachet of approximately 50 mm height × 25 mm width and then placed inside a sealed Al can, maintaining a flat-plate geometry. These measurements were done in reflection geometry, and the presence of the neutron-absorbing ^10^B thus mitigates possible multiple scattering effects related to the increased thickness of the sample. All sample preparation was done in a He atmosphere, and the sample cans were sealed in this atmosphere to avoid decomposition of the sample. An elastic fixed window scan (FWS) between 140 and 370 K was collected to determine the temperature dependence of the reorientational dynamics of KB_3_H_8_.

### First-Principles Calculations

First-principles calculations were performed within the plane-wave implementation of the generalized gradient approximation to density functional theory (DFT) using the PWscf package.^26^ The calculation used a Vanderbilt-type ultrasoft potential and the generalized gradient approximation (GGA) with the Perdew–Burke–Ernzerhof (PBE) exchange correlation function. A cutoff energy of 544 eV and a 4 × 4 × 4 *k*-point mesh (generated by using the Monkhorst–Pack scheme) were enough for the total energy to converge within 0.01 meV per atom. Phonon calculations were performed on the DFT-optimized crystal structure^10^ by using the supercell method (2 × 2 × 2 cell size) with finite displacements.^27^ The simulated INS spectrum was generated within the incoherent approximation, with instrumental resolution taken into account.

## III Results

### Temperature Evolution of the KB_3_H_8_ Polymorphs

**Figure 2:**
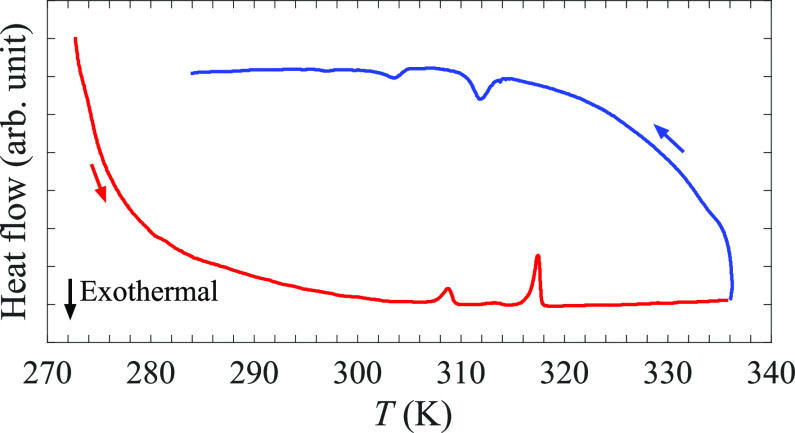
DSC data for KB_3_H_8_. The red curve is measured on heating, while the blue curve is measured on cooling with rates of ±1 K/min.

DSC data of KB_3_H_8_ covering the low-temperature monoclinic (α), orthorhombic (α′), and high-temperature cubic (β) polymorphs are presented in Figure 2. The data suggest that two first-order polymorphic transitions occur during heating, one at 309 K and the other at 316 K, which compare well with the DSC data presented in ref (10). The second-order polymorphic transition from α-KB_3_H_8_ to α′-KB_3_H_8_ occurs at 288 K; thus, it cannot account for the first-order polymorphic transition observed at 309 K. This mismatch in transition order and temperature suggests that an intermediate polymorph exists in between α′-KB_3_H_8_ and β-KB_3_H_8_. This polymorph will hence forward be termed γ-KB_3_H_8_. The transition at 316 K is identified as the γ-to-β transition and is in good agreement with the transition temperature of β-KB_3_H_8_ reported in previous studies.^10,28^

**Figure 3:**
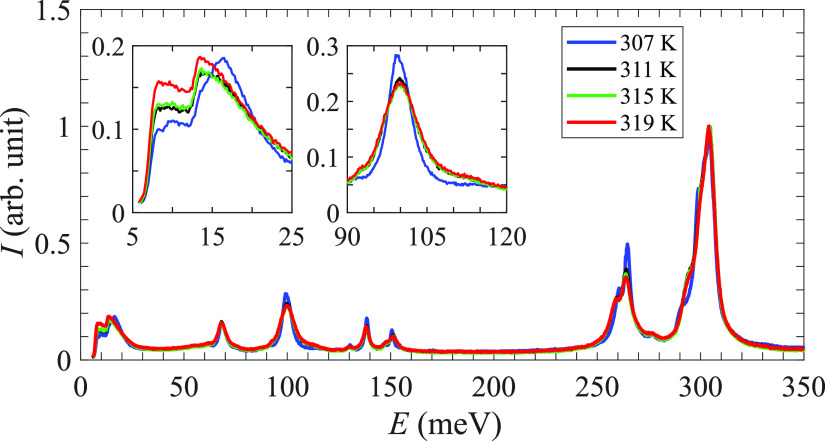
Raman spectra of KB_3_H_8_ at 307, 311, 315, and 319 K. The intensities of the spectra have been normalized to the peak intensity of the strong peak close to 300 meV. The two insets show the data in the limited energy ranges of 5–25 meV and 90–120 meV.

To further study the evolution of the KB_3_H_8_ polymorphs, Raman spectra were collected at several temperatures and are presented in Figure 3. While there are differences between the different spectra, there are large similarities suggesting that only small changes to the local environment upon transitioning between the polymorphs occur. A main difference of the spectra is the broadening that can be observed upon heating, implying an increasing degree of disorder upon entering the γ-polymorph at 311 K. Furthermore, looking at the low-energy part of the spectra (below 25 meV), one can observe a shift in the peak position between the 307 and 311 K spectra and a jump in the peak intensity around 10 meV between the 315 and 319 K spectra, which is in good agreement with the transition temperatures determined by DSC.

**Figure 4:**
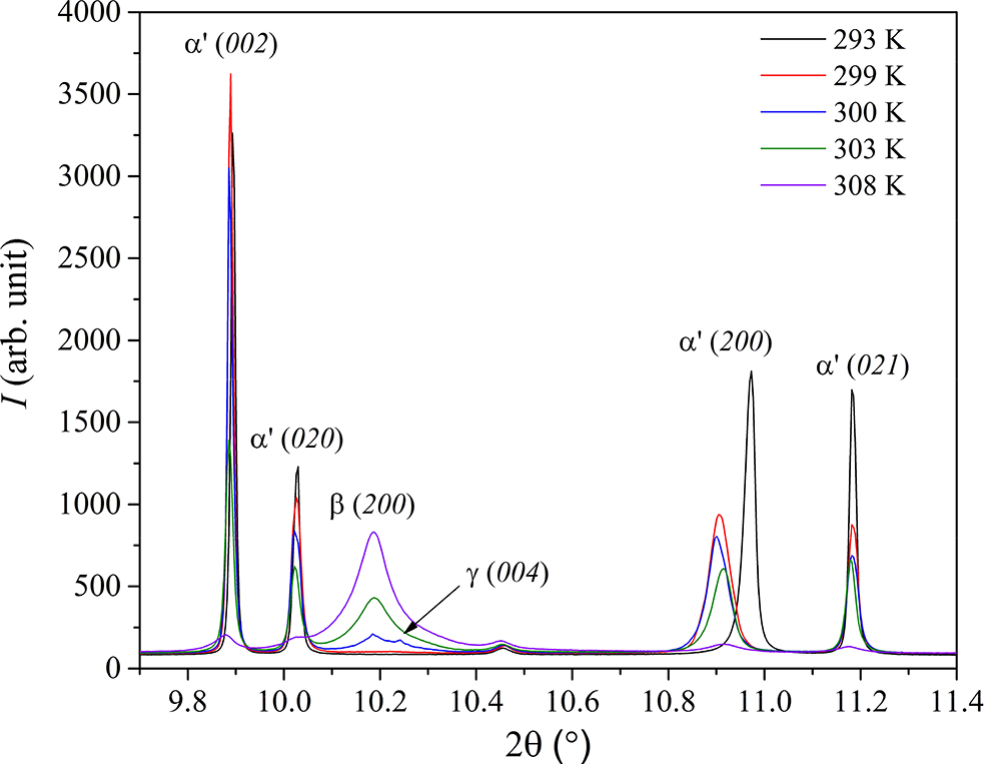
PXD of KB_3_H_8_ in the temperature range of 293–306 K with λ = 0.70870 Å.

To obtain further information about the previously unidentified γ-KB_3_H_8_, an *in situ* SR PXD experiment was conducted in the temperature range 293–328 K, but with a slower heating rate (1 K/min) than in ref (10). The data are presented in Figure S1 of the Supporting Information and agree well with the 5 K/min data presented in ref (10) where a transition from α′-KB_3_H_8_ to β-KB_3_H_8_ is observed at *T* ≈ 303 K. However, a careful inspection of the diffraction patterns, as shown in Figure 4, reveals a significant shift of the (200) reflection in α′-KB_3_H_8_ in the temperature range 293–299 K, corresponding to an expansion of the *a*-axis by 0.04 Å and an increase in the sandwiching K–K distance. At 300 K, the Bragg reflections from α′-KB_3_H_8_ decrease in intensity, and new reflections appear. These reflections can be indexed in a tetragonal unit cell with unit cell parameters *a* = *b* = 7.996 Å and *c* = 15.918 Å and likely correspond to the new γ-polymorph observed by DSC and Raman spectroscopy. γ-KB_3_H_8_ is only observed in a narrow temperature interval; the (004) reflection merges with the (200) reflection upon further heating, and the Bragg reflections can be described by the cubic β-polymorph with unit cell parameters *a* = *b* = *c* = 7.981 Å at *T* = 308 K. The poor crystallinity and broad peaks of γ-KB_3_H_8_ prevent further structural elucidation, but the unit cell parameters suggest that it is similar to α′-KB_3_H_8_, but with similar K–K distances in two directions, and shorter along one axis, the latter likely corresponding to the two K^+^ cations that are “sandwiching” the [B_3_H_8_]^−^ anions.

### Inelastic Neutron Scattering

**Figure 5:**
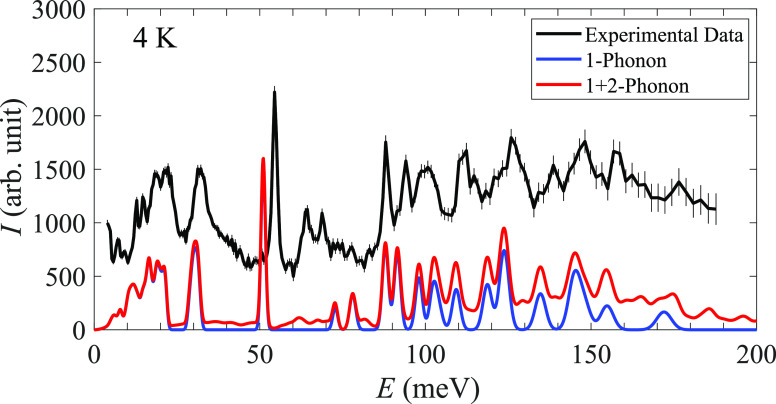
KB_3_H_8_ INS spectra at 5 K compared to the simulated 1-phonon and (1 + 2)-phonon spectra determined from DFT calculations of α-KB_3_H_8_. Error bars correspond to one standard deviation.

Figure 5 shows the INS spectra of KB_3_H_8_ as measured at 4 K in the energy range 5–180 meV together with the calculated spectra. As can be observed, there is a good agreement between the calculated and measured spectra, implying that the structural model presented in ref (10) accurately describes the low-temperature α-polymorph. Based on the calculations, the spectra may be divided into three energy regions: below 25 meV, which contains the librational modes of the [B_3_H_8_]^−^ anion, 25–150 meV, which primarily contains various deformation motions of the anion, and the region above 150 meV, which contains a mixture of bending and stretching motions of the anion. For a more detailed description of the spectra, the reader is referred to the Supporting Information for a list of the vibrational excitation energies as well as animations of the corresponding motions.

### Quasielastic Neutron Scattering

**Figure 6:**
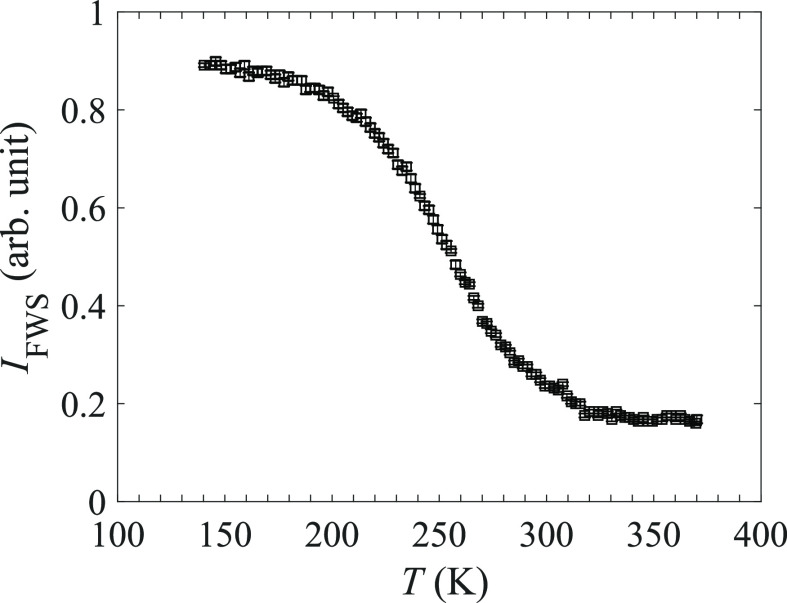
Elastic FWS upon heating from 140 to 370 K with a heating rate of 0.5 K/min. Error bars correspond to one standard deviation.

Figure 6 presents FWS data in the temperature range 140–370 K. During an FWS experiment, the intensity of the elastic peak (a narrow energy slice centered around the elastic peak position) is integrated. As the sample is heated from base temperature, the [B_3_H_8_]^−^ reorientational dynamics will start to develop, which leads to a decrease in the integrated intensity, since the quasielastic component becomes broader than the elastic peak and the total scattering intensity becomes distributed over an energy window larger than the narrow energy slice, leading to a drop in the FWS intensity. Once the dynamics becomes much faster, which is reflected by an ever broader quasielastic line width than the energy slice window, no further decrease in the FWS intensity will be observed, since any intensity loss in the slice window due to further broadening will be minute. At 140 K, the anion reorientational relaxation (residence) times are longer than the accessible experimental time scales of HFBS (τ ≈ 1 × 10^–8^–1 × 10^–11^ s), and only a small decrease is seen between 140 and 200 K, suggesting that the decrease in intensity is mainly related to an increase of the Debye–Waller factor. At ∼200 K, the intensity starts to fall more rapidly with increasing temperature, suggesting that reorientational dynamics starts to occur on the HFBS time scale. The dynamics continues to develop as the temperature is increased further, leading to a continuous drop in the FWS intensity. Above 300 K, the reorientational dynamics starts to become more rapid than the accessible experimental frequency scale of HFBS, and the change in FWS intensity therefore flattens out. This indicates that the relaxation times, τ, of the reorientational dynamics are shorter than 1 × 10^–11^ s.

To study the reorientational dynamics of α-KB_3_H_8_ and α′-KB_3_H_8_, QENS spectra were collected by using HFBS at 235, 253, 273, and 297 K, which is described by the scattering function *S*(*Q*,ω):
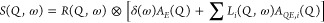
1where *Q* is the momentum transfer of the neutron, *E* = ℏω is the energy transfer of the neutron, ω is the angular frequency, *ℏ* is the reduced Planck constant, *R* is the instrumental resolution function, δ is a delta function, *L*_*i*_ is a Lorentzian function corresponding to index *i*, and *A*_*E*_ and *A*_*QE*_ are the areas corresponding to the respective delta and Lorentzian functions.

**Figure 7:**
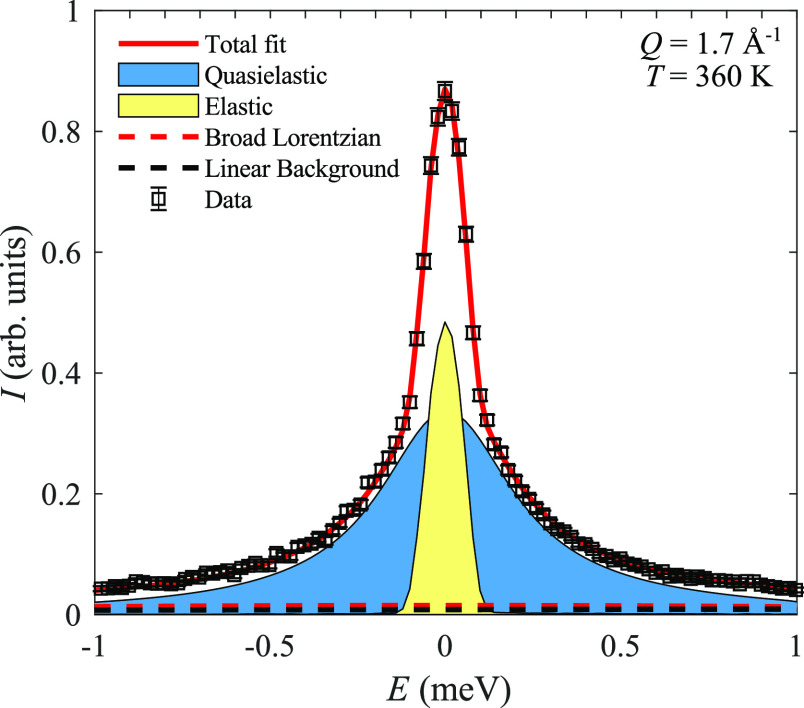
A fit of the QENS data at 360 K and 1.7 Å^–1^, showing the experimental data, the total fit, and the individual components of the fit (eq 1). Error bars correspond to one standard deviation.

The data could be fitted accurately by using eq 1 with a single Lorentzian, implying that only one relaxation time is present at these temperatures. Over the studied temperature interval, the relaxation time develops continuously from around 5 ns (235 K) to 0.5 ns (297 K), which was estimated by using the relation 2*ℏ*/Γ, where Γ is the full width at half-maximum of the Lorentzian. For studies of the γ- and β-polymorphs, QENS spectra were collected by using DCS with a neutron wavelength of 4.8 Å at 311, 320, 339, 360, and 383 K. The QENS spectra for the γ-polymorph (311 K) required one additional Lorentzian, indicating that at least two relaxation times are present. The relaxation time at 311 K is significantly shorter, around 15 ps, compared to the relaxation times of the α-polymorph. Similar to the γ-polymorph, the β-polymorph also requires two Lorentzians. The relaxation time evolves from about 6 ps (320 K) to around 3 ps (383 K) and is thus significantly shorter than the α-polymorph but similar to the γ-polymorph. We note that the spectra for the β-polymorph also require a very broad Lorentzian background to describe the data (see Figure 7). This is expected based on many related QENS studies on the disordered polymorphs of polyhedral borohydrides. Such a broad Lorentzian reflects a more localized, order-of-magnitude more rapid reorientational “rattling” of the [B_3_H_8_]^−^ anion around its equilibrium position.^29,30^

**Figure 8:**
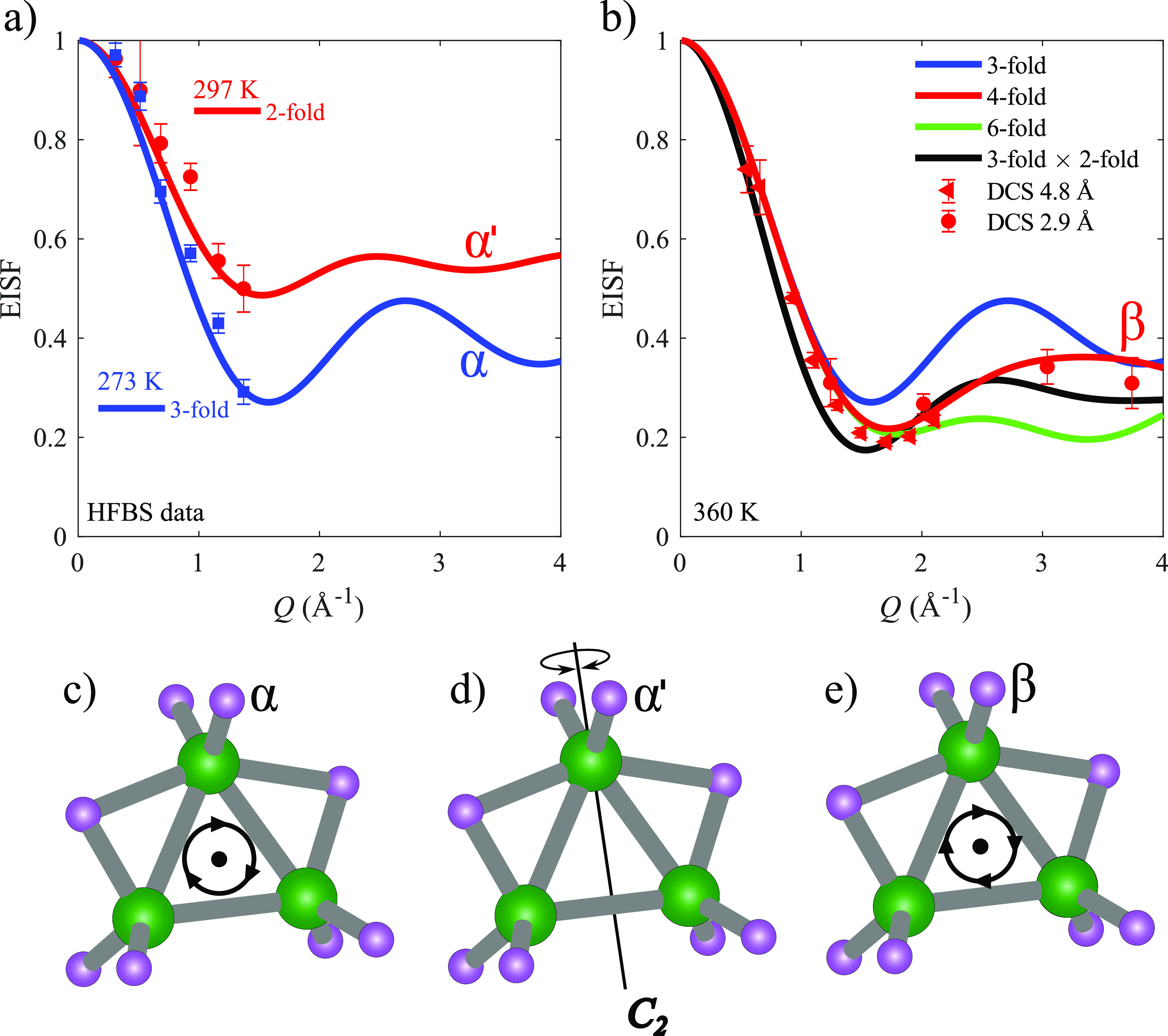
(a) EISF as a function of *Q* for the α-polymorph (blue squares) and α′-polymorph (red circles). The blue line corresponds to an EISF model curve for a 3-fold rotation in the plane created by the three boron atoms. The red line corresponds to a 2-fold rotation around the *C*_2_-axis. (b) EISF as a function of *Q* for the β-polymorph at 360 K for two different incident neutron wavelengths, 4.8 and 2.9 Å. The three lines (blue, red, and green) shown in the figure correspond to 3-, 4-, and 6-fold rotations in the plane created by the three boron atoms, respectively. All of the EISF model curves include an extra contribution of 10% elastic scattering; this extra contribution is assumed to stem from impurities in the sample and the Al sample can. (c, d, e) Schematic representations of the reorientational motions of α-KB_3_H_8_, α′-KB_3_H_8_, and β-KB_3_H_8_, respectively. (c) 3-fold rotations in the plane created by the three boron atoms. (d) 2-fold rotation around the *C*_2_-axis. (e) 4-fold rotations in the plane created by the three boron atoms. Color scheme: B (green) and H (purple). Error bars correspond to one standard deviation.

To determine the reorientational mechanism in KB_3_H_8_, the elastic incoherent structure factor (EISF) as a function of *Q* was extracted from the QENS spectrum at 273, 297, and 360 K. The experimental EISF is defined as EISF(*Q*) = *A*_*E*_(*Q*)/(*A*_*E*_(*Q*) + *A*_*QE*_(*Q*)), where *A*_*E*_ and *A*_*QE*_ correspond to the total integrated elastic and quasielastic scattering, respectively. The experimental EISF and corresponding EISF model curves for different reorientational motions are presented in Figure 8. For the α-polymorph (273 K), the best agreement is found between the data and the model for 3-fold rotation of the [B_3_H_8_]^−^ anion in the boron plane (see Figure 8c). While the data also agree well with a 4-fold rotation around the *C*_2_-axis, this model can be excluded, since a 4-fold rotation would introduce boron partial occupancies, which are not observed by X-ray diffraction (see Figure 1a). A 3-fold reorientation implies partial hydrogen occupancies within this temperature range, which is not in agreement with the structural symmetry presented in Figure 1a. The results thus suggest the need for a neutron diffraction experiment at appropriate temperatures to accurately determine the hydrogen positions in α-KB_3_H_8_. Upon further heating to 297 K, the EISF changes so that it is better described by a 2-fold rotation around the *C*_2_-axis (see Figure 8d). The change in the reorientational motion is reflected by the change in the local environment for the [B_3_H_8_]^−^ anion due to the polymorphic transition from α-KB_3_H_8_ to α′-KB_3_H_8_, which leads to an alignment of the *C*_2_-rotational axis of the [B_3_H_8_]^−^ anion with a K–K axis and an equalization of the distance of the K^+^ cations “sandwiching” the [B_3_H_8_]^−^ anion (see Figure 1d,e). Comparing the experimental EISF of the β-polymorph (360 K) with the model EISF curves, the best agreement is found for a model with 4-fold rotations in the plane formed by the three boron atoms (see Figure 8b,e). However, a rotation in the boron plane does not agree with the reported crystal structure of β-KB_3_H_8_, which suggest a spherical distribution of the [B_3_H_8_]^−^ anion.^10^ Furthermore, rotations in a single specific plane conflict with the cubic crystal structure, which exhibits three equivalent 4-fold reorientation planes. This suggests that the local structure deviates from the global cubic crystal structure on QENS time scales; that is, the local structure distorts slightly so that a single favorable plane of rotation is temporarily created for each individual anion. To shed further light upon this, the crystal structure of β-KB_3_H_8_ is reexamined.

### Crystal Structure of β-KB_3_H_8_

**Figure 9:**
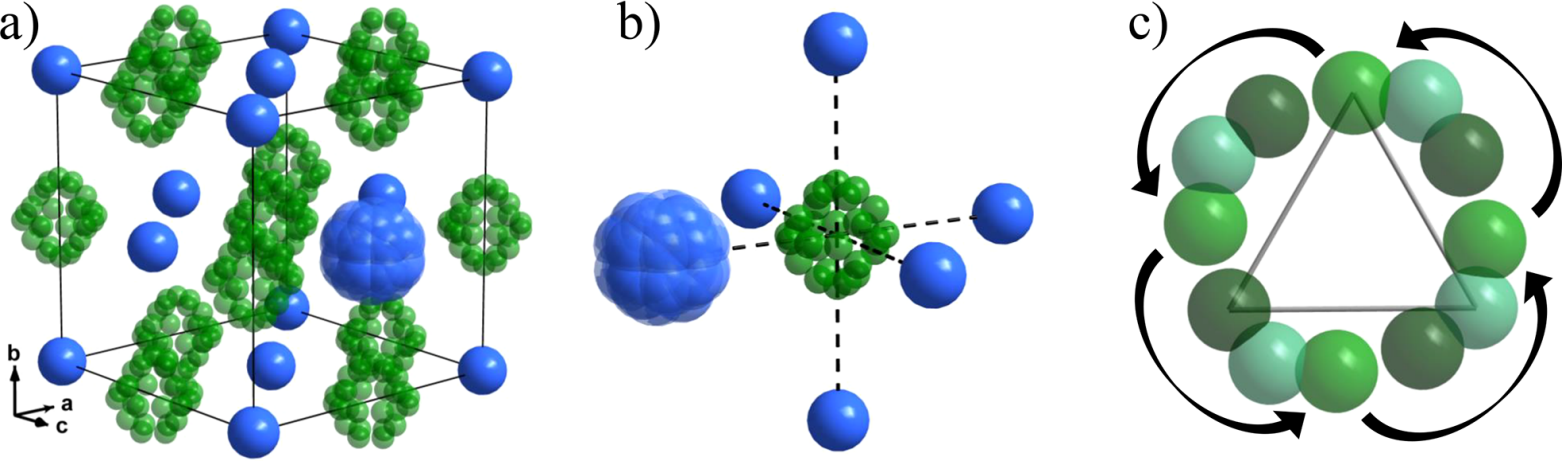
(a) Revised crystal structure of β-KB_3_H_8_, (b) the local environment of the [B_3_H_8_]^−^ anion in the global structure, and (c) the B-positions formed from 4-fold reorientation of the [B_3_H_8_]^−^ anion. K^+^ and [B_3_H_8_]^−^ are positionally disordered around crystallographic sites, shown as transparent atoms. Color scheme: K (blue) and B (green). H atoms are omitted for clarity.

Rietveld refinements of the previously reported β-KB_3_H_8_ resulted in high atomic displacement parameters for both K^+^ and the [B_3_H_8_]^−^ anion to adequately describe the diffraction pattern, which can indicate a significant local distortion of the global structure.^10^ On the basis of the QENS results, we propose a new crystal structure for β-KB_3_H_8_, which is shown in Figure 9a. To adequately model the 4-fold reorientation suggested by QENS, the [B_3_H_8_]^−^ anion is described with partially occupied B and H positions, which results in 12 B positions in each plane of reorientation. Because of the face-centered cubic symmetry, this results in a superposition of three circularly arranged [B_3_H_8_]^−^ units along each crystallographic axis as shown in Figure 9b,c. Rietveld refinements suggest a significant distribution of the K^+^ positions, which is expected as the K–B_3_H_8_ distance in the local structure will depend on the specific orientation of [B_3_H_8_]^−^ anion. To model this, the K atomic positions are refined around the 4a Wyckoff site with partial occupancy, which reveals that a distribution of K^+^ positions exist along the crystallographic axes toward the [B_3_H_8_]^−^ anion. Rietveld refinements result in an improved fit of the diffraction data compared to the previously reported spherically disordered model where high atomic displacement parameters were used.^10^ The Rietveld refinement of β-KB_3_H_8_ is presented in Figure S2.

### Reorientational Energy Barrier

**Figure 10:**
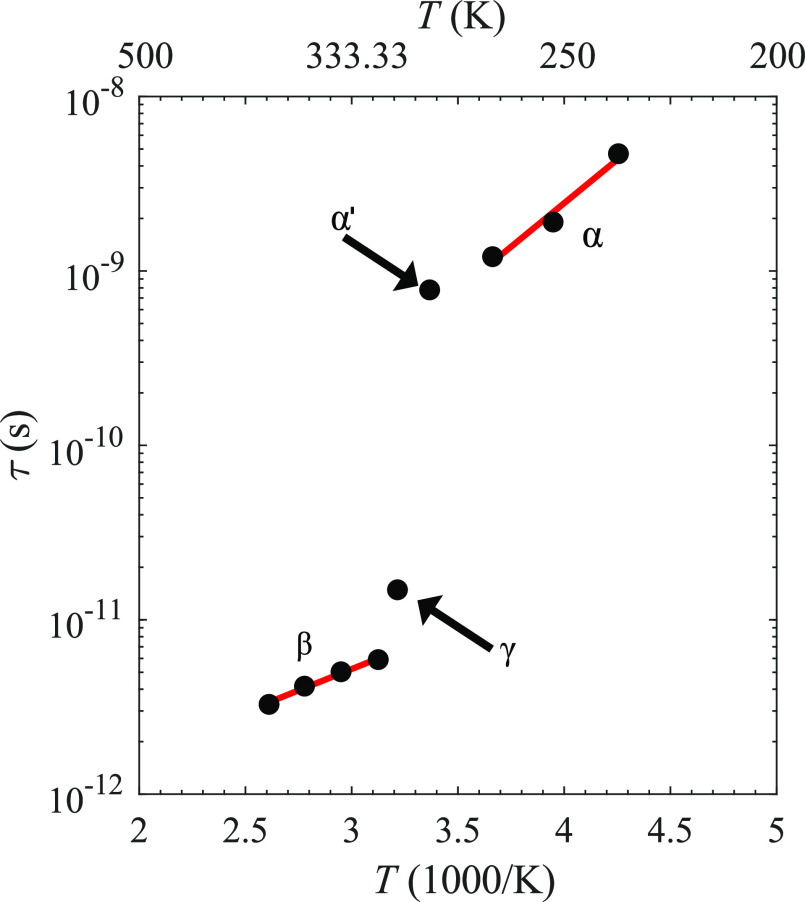
τ extracted from the QENS spectra for the α-, α′-, and β-polymorphs as well as the γ-polymorph. The red lines are linear regressions of the data for α-KB_3_H_8_ and β-KB_3_H_8_.

To determine the energy barrier of the reorientational dynamics, the relaxation times at different temperatures (235, 253, and 273 K for α and 320, 339, 360, and 383 K for β) were fitted to the Arrhenius equation: τ = , where *E*_B_ is the energy barrier, τ_0_ is a prefactor, and *k*_B_ is the Boltzmann constant. The fits and data are presented in Figure 10. The energy barrier of the low-temperature monoclinic α-polymorph was determined to be 199 meV (τ_0_ ≈ 2.5 × 10^–13^), while a barrier of 99 meV (τ_0_ ≈ 1.7 × 10^–13^) was found for the high-temperature cubic β-polymorph. As can be seen in Figure 10, the relaxation time changes drastically at around 297 and 311 K, which coincides with a several-orders-of-magnitude increase of the ionic conductivity of the material.^10^ The simultaneous increase of the conductivity and the decrease of the relaxation time suggest that the anion reorientations play a vital role in promoting the cation transport by flattening the potential energy landscape as reported for M_2_B_*x*_H_*x*_ and MCB_*x*–1_H_*x*_ (M = Li and Na, *x* = 10 and 12)^31,32^ and more recently for KCB_11_H_12_.^29^ With respect to this latter material, both KB_3_H_8_ and KCB_11_H_12_ possess identical cubic disordered structures, rapidly reorienting anions, and full K^+^ cation occupation of the octahedral interstices. One key difference that one might expect to significantly impact the ultimate cation conductivity is the relative sizes of the anions, which directly affects the stable unit cell size. From simple steric arguments, assuming spherical anions with the cubic-close-packed lattice constants for KB_3_H_8_ and KCB_11_H_12_ (≈ 8.017 Å^10^ and ≈10.150 Å,^29^ respectively), one can show that the octahedral and tetrahedral interstices tend to be larger for KCB_11_H_12_ than for KB_3_H_8_, which in effect affords more room for cation translations and is consistent with the roughly 4-orders-of-magnitude higher conductivity observed at 373 K for disordered KCB_11_H_12_ than for KB_3_H_8_. Yet, we do acknowledge that other factors such as the larger deviation from a spherical shape of the B_3_H_8_^–^ anion and the differences in anion reorientational mobilities and mechanisms may also be affecting the conductivities in unknown ways.

## IV Conclusions

KB_3_H_8_ has been investigated by using differential scanning calorimetry, Raman spectroscopy, *in situ* synchrotron radiation powder X-ray diffraction, inelastic neutron scattering, first-principles calculations, and quasielastic neutron scattering. The results revealed the existence of a previously unidentified polymorph in between the previously reported α′- and β-polymorphs. Furthermore, the results highlight the connection between the local structure and the reorientational dynamics of the [B_3_H_8_]^−^ anion and suggest, that on a local level, the crystal structure of β-KB_3_H_8_ is distorted and deviates from the cubic global structure. Over the studied temperature interval, 235–383 K, the reorientational motion changes from a 3-fold rotation in the boron plane in α-KB_3_H_8_ to a 2-fold rotation around the *C*_2_-axis in α′-KB_3_H_8_, and into a 4-fold rotation in the boron plane for β-KB_3_H_8_. Based on these results, a new crystal structure for β-KB_3_H_8_ was proposed, which better describes the disorder of the [B_3_H_8_]^−^ anion. Furthermore, the findings suggest that studies of the local order, using for example total scattering techniques, would be beneficial to fully understand the properties of KB_3_H_8_. Finally, the results imply that the fast reorientations of the [B_3_H_8_]^−^ anion play an important role in the cation diffusion, similar to what has been observed for Li, Na, and K polyhedral closo- and nido- (carba)borates.^29,31−33^

## Supporting Information Available

Information about the *in situ* SR PXD experiment, the Rietveld refinement of β-KB_3_H_8_, and the vibrational excitation energies and animations for α-KB_3_H_8_ (PDF)
File containing the vibrational excitaion energies and animations for α-KB_3_H_8_ (ZIP)

## Notes

The crystalographic data of β-KB_3_H_8_ is available at www.ccdc.cam.ac.uk using the identifier CCDC 2056973.
