# Supplementary material for: Interplay between the Reorientational Dynamics of the B3H8– Anion and the Structure in KB3H8
Source: J Phys Chem C Nanomater Interfaces. 2021 Feb 16;125(7):3716–24. doi: 10.1021/acs.jpcc.0c10186 (PMC8023716; doi:10.1021/acs.jpcc.0c10186)
Supplement: Supplementary file 1 — jp0c10186_si_002.pdf [file jp0c10186_si_002.pdf]

Supporting Information for:

Interplay Between the Reorientational Dynamics of the $B_3H_8^-$ Anion and the Structure in KB_3H_8

M. S. Andersson,[1,2,*] J. B. Grinderslev,[3] X.-M. Chen,[4] X. Chen[4,5] U. Häussermann,[6] W. Zhou,[2] T. R. Jensen,[3] M. Karlsson[1] and T. J. Udovic[2,7]

[1]*Department of Chemistry and Chemical Engineering, Chalmers University of Technology, Göteborg SE-412 96, Sweden*
E-mail: anmikael@chalmers.se (M. S. Andersson).

[2]*NIST Center for Neutron Research, National Institute of Standards and Technology, Gaithersburg, Maryland 20899-6102, United States*

[3]*Interdisciplinary Nanoscience Center (iNANO), Center for Energy Materials, and Department of Chemistry, Aarhus University, DK-8000, Denmark*

[4]*School of Chemistry and Chemical Engineering, Henan Key Laboratory of Boron Chemistry and Advanced Energy Materials, Henan Normal University, Xinxiang, Henan 453007, China*

[5]*College of Chemistry and Green Catalysis Center, Zhengzhou University, Zhengzhou, Henan 450001, China*

[6]*Department of Materials and Environmental Chemistry, Stockholm University, SE-10691 Stockholm, Sweden*

[7]*Department of Materials Science and Engineering, University of Maryland, College Park, MD 20742-2115, United States*

1 In situ SR PXD data of KB_3H_8

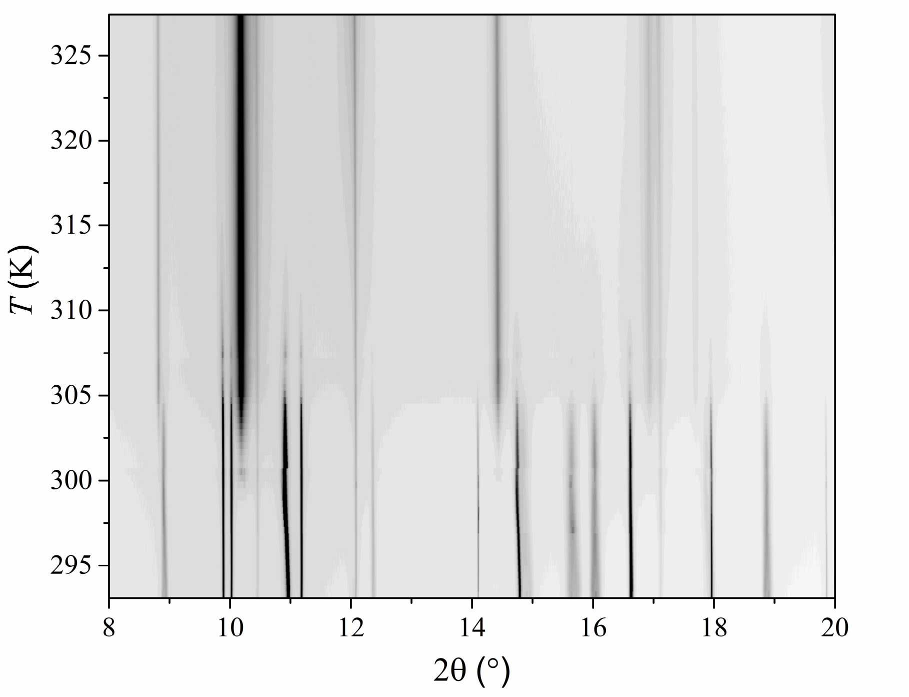

Fig. S1 In situ SR PXD data of KB_3H_8 in the temperature range T = 293–328 K with a heating rate of 1 K/min and λ = 0.70870 Å

2 Rietveld refinement of β-KB_3H_8

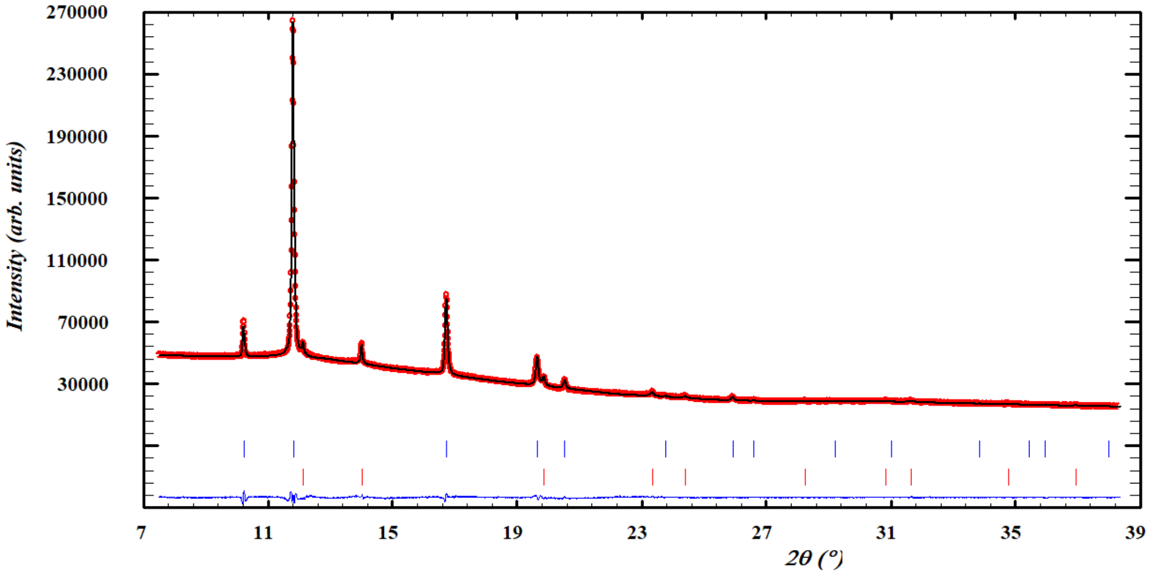

Fig. S2 Rietveld refinement plot of SR PXD data measured at 50 °C, λ = 0.824598 Å, for β-KB_3H_8, showing experimental (red circles) and calculated (black line) SR PXD patterns, and a difference plot below (blue). Top tick (β-KB_3H_8, *Fm-3m*), bottom tick (KBH_4, *Fm-3m*). Final discrepancy factors: R_p = 0.914 %, R_{wp} = 1.26 % (not corrected for background), R_p = 17.5 %, R_{wp} = 8.15 % (conventional Rietveld R-factors), R_{Bragg}(β-KB_3H_8) = 3.28 % and global χ^2 = 4.65.

3 Phonon modes

A .ascii file is provided as part of the SI. It contains the information needed to view the animated (gamma-point) phonon normal modes from the DFT-optimized structure, and can be opened using the open source software V_Sim (https://gitlab.com/l_sim/v_sim). N.B., the use of this software does not imply its recommendation or endorsement by NIST.
